# Draft Genome Sequence of Herminiimonas contaminans Strain CCM 7991^T^, a Biopharmaceutical Contaminant

**DOI:** 10.1128/MRA.01432-20

**Published:** 2021-02-18

**Authors:** Luis Johnson Kangale, Rita Zgheib, Eric Ghigo, Didier Raoult, Pierre-Edouard Fournier

**Affiliations:** a Aix-Marseille University, IRD, AP-HM, SSA, VITROME, Marseille, France; b IHU Méditerranée Infection, Marseille, France; c Aix-Marseille University, IRD, AP-HM, MEPHI, Marseille, France; d Special Infectious Agents Unit, King Fahd Medical Research Center, King Abdulaziz University, Jeddah, Saudi Arabia; e Techno-Jouvence, Marseille, France; University of Southern California

## Abstract

Herminiimonas contaminans was described as a new bacterial species, a contaminant isolated from a biopharmaceutical production process in Sweden. Since the genome sequence was not available, we performed draft genome sequencing. The genome of strain CCM 7991^T^ (=CCUG 53591^T^ = DSM 28178^T^ = Marseille-Q4544^T^) was 4,038,814 bp long, with a G+C content of 53.9%; a total of 3,860 genes were identified, along with 3 rRNAs, 44 tRNAs, and 4 noncoding RNAs (ncRNAs).

## ANNOUNCEMENT

The genus *Herminiimonas* was first described by Fernandes et al. in 2005, with Herminiimonas fonticola as the type species (1). Strain CCM 7991 is a contaminant isolated from a biopharmaceutical production process in Sweden and was described as the type strain of Herminiimonas contaminans (2). Working on the planarian microbiota, we identified a new bacterial species. The characterization of this species required a comparison with *H. contaminans*, but no genome sequence was available for *H. contaminans* at the time. We acquired *H. contaminans* DSM 28178^T^ (=CCM 7991^T^) from the Deutsche Sammlung von Mikroorganismen und Zellkulturen GmbH (DSMZ). *H. contaminans* DSM 28178^T^ was then deposited in the Collection des Souches de l’Unité des Rickettsies (CSUR) culture collection under the number Marseille-Q4544^T^. However, because no genome sequence was available for *H. contaminans*, we performed whole-genome sequencing for this species. In our laboratory, strain Marseille-Q4544^T^ was grown on Columbia agar supplemented with 5% sheep blood (bioMérieux, Marcy l’Etoile, France), at 28°C for 24 h. To extract bacterial genomic DNA (gDNA) from strain Marseille-Q4544^T^, a mechanical treatment was first performed on a single colony by glass beads acid washed (Sigma-Aldrich Chimie, Saint-Quentin-Fallavier, France) using a FastPrep-24 5G grinder (MP Biomedicals, Illkirch, France) at maximum speed (6.5) for 90 sec. Then, after a 30-min lysozyme incubation at 37°C, DNA was extracted on an EZ1 BioRobot system with the EZ1 DNA tissue kit (Qiagen, Hilden, Germany). The gDNA was sequenced using MiSeq technology (Illumina, Inc., San Diego, CA, USA) (3) with the Nextera XT DNA sample prep kit (Illumina) and mate pair strategy. The gDNA was fragmented and then amplified by 12 cycles of PCR, followed by the addition of tag adapters and the introduction of dual-index barcodes. The libraries were purified using AMPure XP beads (Beckman Coulter, Inc., Fullerton, CA, USA), normalized using specific beads following the Nextera XT protocol, and pooled into a single library for sequencing. After 39 h, automated cluster generation and paired-end sequencing with dual-index reads were performed in a single run in 2 × 250-bp format. Total information of 5.51 Gb was obtained from a 578,000/mm^2^ cluster density with clusters passing quality control filters at 93.8%. Within this run, the index representation for strain Marseille-Q4544^T^ was determined to be 6.2%. The 11,232,685 paired-end reads of the MiSeq run were quality checked using FastQC version 0.11.8 with default parameters (4). We used Shovill on the Galaxy Australia server (www.usegalaxy.org.au) (Galaxy version 1.0.4+galaxy0) with the following options: Trimmomatic version 0.39 for trimming the reads and SPAdes version 3.14 for genomic assembly, with default parameters (5, 6). Strain Marseille-Q4544^T^ was assembled into 56 contigs (*N*_50,_ 201,954 bp; *L*_50_, 6; coverage, 46.95×) for a total size of 4,038,814 bp, with a G+C content of 53.9%. Genomic annotation was obtained using the NCBI Prokaryotic Genome Annotation Pipeline (PGAP) version 4.13 (7). A total of 3,860 genes were identified, along with 3 rRNAs, 44 tRNAs, and 4 noncoding RNAs (ncRNAs). A BLASTN search of complete 16S rRNA gene sequences on NCBI showed 100% identity with Herminiimonas contaminans (16S rRNA GenBank accession number NR_108871.1)

### Data availability.

The genome and read sequences of Herminiimonas contaminans strain Marseille-Q4544^T^ (BioProject PRJNA677876 and BioSample SAMN16775933) were deposited in GenBank under the accession numbers JADOEL010000001 through JADOEL010000056 and SRR13040143, respectively. Herminiimonas contaminans strain Marseille-Q4544^T^ is available in the CSUR, CCM, CCUG, and DSMZ culture collections under the accession numbers CSUR Q4544^T^, CCM 7991^T^, CCUG 53591^T^, and DSM 28178^T^, respectively.
